# Sexual selection, environmental robustness, and evolutionary demography of maladapted populations: A test using experimental evolution in seed beetles

**DOI:** 10.1111/eva.12758

**Published:** 2019-02-19

**Authors:** Ivain Martinossi‐Allibert, Emma Thilliez, Göran Arnqvist, David Berger

**Affiliations:** ^1^ Department of Ecology and Genetics, Animal Ecology Uppsala University Uppsala Sweden

**Keywords:** adaptation, environmental change, maladaptation, population viability, sexual conflict, sexual reproduction, sexual selection, stress

## Abstract

Whether sexual selection impedes or aids adaptation has become an outstanding question in times of rapid environmental change and parallels the debate about how the evolution of individual traits impacts on population dynamics. The net effect of sexual selection on population viability results from a balance between genetic benefits of “good‐genes” effects and costs of sexual conflict. Depending on how these facets of sexual selection are affected under environmental change, extinction of maladapted populations could be either avoided or accelerated. Here, we evolved seed beetles under three alternative mating regimes to disentangle the contributions of sexual selection, fecundity selection, and male–female coevolution to individual reproductive success and population fitness. We compared these contributions between the ancestral environment and two stressful environments (elevated temperature and a host plant shift). We found evidence that sexual selection on males had positive genetic effects on female fitness components across environments, supporting good‐genes sexual selection. Interestingly, however, when males evolved under sexual selection with fecundity selection removed, they became more robust to both temperature and host plant stress compared to their conspecific females and males from the other evolution regimes that applied fecundity selection. We quantified the population‐level consequences of this sex‐specific adaptation and found evidence that the cost of sociosexual interactions in terms of reduced offspring production was higher in the regime applying only sexual selection to males. Moreover, the cost tended to be more pronounced at the elevated temperature to which males from the regime were more robust compared to their conspecific females. These results illustrate the tension between individual‐level adaptation and population‐level viability in sexually reproducing species and suggest that the relative efficacies of sexual selection and fecundity selection can cause inherent sex differences in environmental robustness that may impact demography of maladapted populations.

## 1 INTRODUCTION

Evolutionary rescue critically depends on genetic responses being rapid enough to allow populations to track changes in their environment while the demographic cost of maladaptation remains small enough to avoid genetic drift and extinction (Bell & Gonzales, 2009, Carlson, Cunningham, & Westley, 2014; Derry, 2019; Gonzalez, Ronce, Ferriere, & Hochberg, 2013; Orr & Unckless, 2014; Walters, Blanckenhorn, & Berger, 2012). Research has further highlighted the potential discrepancy between adaptation in individual traits and that of the population as a whole (Bolnick et al., 2011; Cam, Link, Cooch, Monnat, & Danchin, 2002; Rankin, Dieckmann, & Kokko, 2011; Svensson & Connallon, 2019; Violle et al., 2007). This discrepancy may have fundamental influence on the potential for evolutionary rescue because those strategies that maximize individual fitness may often lead to overexploitation of ecological resources and therefore do not always translate into maximal population viability (Hardin, 1968; Kokko & Brooks, 2003; Rankin & López‐Sepulcre, 2005; Svensson & Connallon, 2019). In sexually reproducing species, these dynamics can become of particular importance, and because while population growth often depends strongly on female egg production, adaptation in traits increasing male fertilization success may have only weak, and sometimes even negative, effects on population viability (Arnqvist & Rowe, 2005; Clutton‐Brock & Parker, 1995; Rankin et al., 2011, see also Fraser, 2019 of this special issue). This realization has sparked considerable debate about whether sexual selection generally will act to increase or decrease extinction risk (Gerber & Kokko, 2016; Hamilton & Zuk, 1982; Holman & Kokko, 2014; Kokko & Brooks, 2003; Li & Holman, 2018; De Lisle, Goedert, Reedy, & Svensson, 2018; Lorch, Proulx, Rowe, & Day, 2003; Manning, 1984; Martínez‐Ruiz & Knell, 2017; Martinossi‐Allibert, Rueffler, Arnqvist, & Berger, 2018; Martinossi‐Allibert, Savković et al., 2018; Maynard‐Smith, 1991; Rankin et al., 2011; Whitlock & Agrawal, 2009; Zahavi, 1975).

Sexual selection in polygamous species often acts through pre‐ and postcopulatory female mate choice or male–male competition based on morphological or behavioral traits such as mating calls, antlers, body size, coloration, or sperm characteristics (Andersson, 1994). Hypotheses suggesting that sexual selection should increase population fitness assume that the expression and maintenance of these traits are energetically costly and therefore reflect the bearer's overall condition and genetic quality (Hamilton & Zuk, 1982; Jennions, Moller, Petrie, Mller, & Bernard, 2001; Zahavi, 1975). Such sexual selection for “good genes” could therefore target large parts of the genome and purge deleterious mutations with pleiotropic effects on survival and female fecundity (the genic capture hypothesis, Rowe & Houle, 1996; Tomkins, Radwan, Kotiaho, & Tregenza, 2004). Moreover, it would do so while leaving females relatively spared of the cost of adaptation, allowing population fitness and viability to remain largely unaffected in the process (Manning, 1984; Whitlock & Agrawal, 2009).

This idea has been contested by studies on a variety of systems that have revealed misalignment of selection in the sexes (reviewed in: Bonduriansky & Chenoweth, 2009; Rice & Gavrilets, 2014). One fundamental consequence of this is that, because males and females share most of their genome, alleles favored in one sex may be detrimental when expressed in the other, resulting in a genetic conflict known as intralocus sexual conflict (IaSC: Rice & Chippindale, 2001). IaSC may thus reduce or even reverse any positive contribution that selection for good genes makes to population viability.

Sexual selection can also have direct detrimental effects at the population level. This can happen if the successful male strategy inflicts harm on the female during the mating interaction, reducing her fecundity or longevity. Indeed, such male strategies have been observed in a wide range of animal taxa (Arnqvist & Rowe, 2005; Clutton‐Brock & Parker, 1995; Parker, 1979, 2006; Thornhill & Alcock, 1983). This form of conflict, referred to as interlocus sexual conflict (IeSC), thus represents a type of “tragedy of the commons,” in which male adaptations can in theory drive a population to extinction by overexploiting the main resource‐limiting population growth (the female and her egg production) (Kokko & Brooks, 2003; Rankin et al., 2011).

These costs and benefits of sexual selection may produce variable outcomes at the population level, as observed in experimental systems providing evidence for sexual selection increasing adaptation (Fricke & Arnqvist, 2007; Grieshop, Stångberg, Martinossi‐Allibert, Arnqvist, & Berger, 2016; Mallet, Bouchard, Kimber, & Chippindale, 2011; Mcguigan, Petfield, & Blows, 2011; Plesnar‐Bielak, Skrzynecka, Prokop, & Radwan, ; Sharp & Agrawal, 2013) as well as impeding it (Arbuthnott & Rundle, 2012; Berger, Martinossi‐Allibert et al., 2016; Chenoweth, Appleton, Allen, & Rundle, 2015; Holland, 2002; Hollis & Houle, 2011; Rundle, Chenoweth, & Blows, 2006). The impact of environmental change on these dynamics has started to be explored in recent years (Arbuthnott, Dutton, Agrawal, & Rundle, 2014; Berger et al., 2014; Connallon & Clark, 2014; Gerber & Kokko, 2016; Gomez‐Llano, Bensch, & Svensson, 2018; Holman & Jacomb, 2017; Li & Holman, 2018; Martinossi‐Allibert, Rueffler et al., 2018; Martinossi‐Allibert, Savković et al., 2018; Parrett & Knell, 2018; Plesnar‐Bielak et al., 2018; Punzalan, Delcourt, & Rundle, 2014; Skwierzyńska, Radwan, & Plesnar‐Bielak, 2018; Yun et al., 2018), and there are indeed reasons to suspect that the different facets of sexual selection will be sensitive to rapid ecological change. For example, male reproductive traits often exhibit genotype‐by‐environment interactions (GEI:s) (Bussiere, Hunt, Stölting, & Jennions, 2008; Kolluru, 2014; Miller & Svensson, 2014). Such changes in genotype‐ranking across environments may simply reflect strong sexual selection favoring locally adapted male genotypes (Martinossi‐Allibert, Arnqvist, & Berger, 2017), but also bring up the question of whether sexual selection generally favors genotypes of high environmental robustness. If sexually selected traits are costly, as predicted by theory, sexual selection could favor resource allocation decisions that under certain circumstances lead to increased mortality in the population (Brooks, 2000; Hunt et al., 2004; Kim & Velando, 2016; Zajitschek & Connallon, 2017). On the other hand, if secondary sexual traits are honest signals of condition (Hamilton & Zuk, 1982; Jennions et al., 2001; Zahavi, 1975), good‐genes sexual selection may instead favor high‐quality genotypes that are resilient to most types of stress. While this question has received attention in studies measuring GEI:s for traits presumably involved in sexual selection (reviewed in: Bussiere et al., 2008; Kolluru, 2014; Miller & Svensson, 2014), experiments directly linking sexual selection to the manifestation of GEI:s and resilience to rapid environmental change are scarce. Moreover, an underappreciated consequence of sex‐specificity in such GEI:s are potential knock‐on effects at the population level; because the extent of male‐induced harm on females should depend on the relative condition of males and females (Clutton‐Brock & Parker, 1995; Parker, 2006; Rankin et al., 2011), IeSC may be modulated in accordance with the sensitivity of each sex to the change in ecological conditions. However, this hypothesis, suggesting that sex differences in environmental tolerance could affect the intensity of IeSC and its demographic consequences, remains largely unexplored (but see Perry & Rowe, 2018). In Supporting Information Figure S1, we outline and detail some of the scenarios for how a history of individual‐level selection acting on fecundity and competitive fertilization success is predicted to affect the viability of maladapted populations facing novel environmental conditions.

Here, we contrasted the contribution of sexual selection to population fitness in well‐adapted populations assayed in their ancestral environment, and when reared on a suboptimal host plant or at elevated temperature, to which the populations were maladapted. To do this, we first subjected replicate lines of seed beetle to experimental evolution under three alternative mating system regimes: Polygamy (allowing sexual selection, fecundity selection, and male–female coevolution), enforced Monogamy (allowing only fecundity selection and minimizing sexual conflict), and Male‐limited selection (allowing only sexual selection on males and prohibiting females to coevolve with males). Following 16–20 generations of experimental evolution, the lines were reared in either the ancestral or one of the two stressful environments and males and females were assayed for their lifetime individual reproductive success in competitive settings. At the ancestral and elevated temperature, we also assayed the beetles’ fertility as monogamous pairs and their joint offspring production in larger groups. These assays allowed us to explore how evolution under alternative mating systems and levels of sexual versus fecundity selection affected individual‐ and population‐level estimates of fitness (as well as the link between them) in an ancestral and novel environment. It also allowed us to quantify IeSC in terms of the net cost of sociosexual interactions. Specifically, we could infer the importance of sexual selection for female fitness by comparing offspring production in male‐limited and polygamous females evolving with sexual selection, to that in monogamous females evolving without it. We could also assess how the opportunity for male–female coevolution affected the impact of sexual conflict on population viability by comparing offspring production in groups of beetles from the polygamous lines (allowing male–female coevolution) and male‐limited lines (where female counteradaptation was prevented). In Table 1, we outline our expectations for how these fitness measures should differ between the three evolution regimes when either “good genes” or sexual conflict is the prevailing effect of sexual selection.

**Table 1:** Predictions for differences among the three selection regimes (P = Polygamy selection, Ma = Male‐limited sexual selection, Mo = Monogamy selection) for the five fitness measures under three main scenarios for the effects of sexual selection

Measure	Good genes	IaSC	IeSC
Male LRS	P > Ma > Mo	Ma > P > Mo	P = Ma>Mo
Female LRS	P > Ma > Mo	Mo > P > Ma	P > Mo>Ma
Fertility	P > Ma > Mo	Mo > P > Ma	Mo > P > Ma
Population fitness	P > Ma > Mo	Mo > P > Ma	P > Mo>Ma
Cost of mating	Ma > P > Mo	Ma > P > Mo	Ma > P > Mo

Note

The “good genes” and intralocus sexual conflict (IaSC) scenarios assume a genetic correlation for fitness, r_MF _= 1 and −1, respectively. The scenario where interlocus sexual conflict (IeSC) is the prevailing effect of sexual selection assumes that r_MF _= 0. Based on empirical data on seed beetles, all scenarios assume that sexual selection in males > fecundity selection in females. Note that the population‐level cost of sociosexual interactions may differ between selection regimes under all three scenarios, given that males and females may evolve differences in their relative abilities to coerce (males) and reject/tolerate (females) matings. Note also that male and female LRS were measured against a standard polygamous reference stock, whereas the other three estimates are “within‐population” measures. The predictions summarized in this table are based on the scenarios described in Supporting Information Figure S1. We test these predictions in well‐adapted and maladapted populations (see Results).

## 2 METHODS

### 2.1 Study population

The seed beetle *Callosobruchus maculatus* is a common pest on fabaceous seeds usually found in tropical and subtropical regions. Larvae develop inside the beans of their host and emerge as reproductively mature adults; during adult life, they do not require food or water to complete their life cycle (Fox, Stillwell, & Wallin, 2011). One of the preferred environments of *C. maculatus*, seed storages in tropical regions, is easy to reproduce in the laboratory, which makes it an ideal model system (Fox, Bush, & Wallin, 2003; Messina & Jones, 2009). All the beetle stocks that were used in this study were maintained under controlled temperature (29°C), humidity (50% RH), and light cycle (12L: 12D) and reared on the preferred host plants *Vigna unguiculata* (black‐eyed bean). Under these conditions, adult lifespan lasts typically between 7 and 14 days.

The base population, on which our three experimental evolution regimes were applied, is a conglomerate of 41 isofemale lines that were isolated from a natural population sampled in Lomé, Togo (06°10#N 01°13#E), in 2010 (see Berger et al., 2014). Creating isofemale lines from the original population allowed us to capture and maintain a snapshot of the genetic variation present in the natural population at the time of sampling (Hoffmann & Parsons, 1988). The isofemale lines were maintained under controlled temperature (29°C), humidity (50% RH), and light cycle (12L: 12D) and were reared on the preferred host plant, *V. unguiculata* (black‐eyed bean). The base population was created by mixing 30 randomly selected individuals from each isofemale line. After two generations of mixing, the large base population (*N* > 3,000) was split into three replicate populations. Each replicate was then split among three evolution regimes that were maintained for 16 generations at the same benign (ancestral) conditions as the isofemale lines prior to the first experiment (see below).

### 2.2 Evolution regimes

To decouple the effects of fecundity selection, sexual selection, and male–female coevolution on individual reproductive success and population fitness, we allowed beetles to evolve under three alternative mating regimes: Polygamy (allowing both fecundity and sexual selection, and male–female coevolution), Monogamy (allowing only fecundity selection on male–female mating pairs), and Male‐limited selection (allowing only sexual selection on males and nullifying selection on females). One of the replicates of the Male‐limited evolution regime was lost due to mishandling during the experimental evolution protocol, which brought the number of replicates used in the present study to three for the Polygamy and Monogamy regimes and two for the Male‐limited selection regime. Effective population size in each regime was kept approximately equal (N_e _= 150) by first estimating the variance in lifetime reproductive success expected for each sex, based on previously published data on this population (Berger et al., 2014; Berger, You et al., 2016; Martinossi‐Allibert et al., 2017), and then using it to estimate the population size necessary to obtain an N_e_ of 150 following the equation provided in Falconer and MacKay (1996):

$${\text{N}}_{\text{e}}\;\text{=}\;\frac{\text{8}N}{\text{vm}+\text{vf}+4}$$

where vm and vf are the variances in reproductive success of males and females.

The number of beans provided as egg‐laying substrate in each regime was standardized to give the same, relatively low, juvenile density (2–4 eggs/bean) to minimize (and equalize) larval competition.

### 2.3 Polygamy

Under the Polygamy regime, both males and females experienced selection and had opportunities to mate multiply; this evolution regime was close to natural conditions or regular laboratory maintenance conditions. Each generation, 300 adults were transferred to a glass jar containing approximately 4,800 black‐eyed beans. During 48 hr, individuals were free to interact, copulate, and lay eggs on the available beans. After 48 hr, adults were removed from the jar and the beans were saved until emergence of the next generation at which point 300 individuals were randomly collected to seed the new generation.

### 2.4 Monogamy

Under the Monogamy regime, virgin individuals were paired at random in monogamous couples in order to remove sexual selection. 0–72 hr postadult emergence, 123 couples were created and each couple was left to interact during 5 hr in a 6‐cm Petri dish, to allow for multiple matings and male–female interactions. After that, all females were gathered in a glass jar containing approximately 4,800 black‐eyed beans and left 48 hr to lay eggs. After 48 hr, females were removed and the beans were saved until the emergence of the next generation. In this regime, selection should have been acting on female fecundity and ability to oviposit on high‐quality substrate (by selecting larger beans or beans presenting low competition, free of eggs laid by other females), as well as on males with positive effects on the fecundity of their female via ejaculate composition and mating behavior.

### 2.5 Male‐limited selection

Under the Male‐limited regime, selection on females was removed while sexual selection was allowed to act in males. Hundred virgin individuals of each sex were placed in a 1‐L glass jar containing a cardboard structure but no beans. This provided a more complex environment than a simple empty jar and allowed individuals to find hiding places (which they normally find among the beans) without having to provide beans on which females would have laid eggs. After 48 hr, during which individuals interacted and copulated at will, females were removed from the jar and placed in individual 6‐cm Petri dishes containing ca. 30 black‐eyed beans, where they were left for 48 hr to lay eggs. The next generation was formed by collecting one random offspring of each sex from each dish. This insured that very weak selection was acting in females because all had the same genetic contribution to the next generation, except for the few females (a maximum of three in any generation) that died before egg laying, whereas sexual selection will have favored the males fertilizing the highest fraction of female eggs.

### 2.6 Experimental design

Competitive lifetime reproductive success (LRS) of individual males and females was measured after 16 generations of experimental evolution, followed by one generation of relaxed selection. Fertility following a single monogamous mating, “population fitness” of mixed‐sex groups, and traits putatively related to reproductive success (body weight, ejaculate weight, locomotor activity) were measured after 20 generations of experimental evolution, and one subsequent generation of relaxed selection. The environmental sensitivity of competitive LRS was assayed in individuals raised as larvae in one of three environments: the ancestral condition (29°C on black‐eyed beans), and two stressful conditions: elevated temperature (36°C, black‐eyed beans) and host plant shift (adzuki beans, *Vigna angularis*, at 29°C). Fertility and population fitness were assayed only at the ancestral and elevated temperature conditions due to logistic limitations. The environmental sensitivity of LRS could be estimated independently in males and females by competing them against a reference stock raised at the ancestral conditions. Fertility and population fitness assays, on the other hand, estimated offspring production for each line as a whole. Moreover, from these latter two estimates, we could quantify the cost of sociosexual interactions (i.e., level of IeSC), by comparing the per female offspring production in the two assays (see further below).

### 2.7 Competitive LRS

Thirty mating pairs were created from each replicate line of the evolution regimes and split equally among the three larval environments, resulting in 10 pairs per environment. These pairs were then allowed to mate and produce offspring. Between 5 and 10 offspring per sex and mating pair were scored for competitive LRS, for a total of 3,672 assays evenly distributed across evolution regimes, sexes, and environments.

Competitive LRS was measured by competing focal males and females from the evolution regimes to a reference population formed some 60 generations previously from the same genetic stock, kept at the same abiotic ancestral lab conditions as the evolution regimes, under the natural polygamous mating regime. A virgin focal individual (raised in one of the three larval environments) was placed in a 9‐cm Petri dish containing a nonlimiting amount of black‐eyed beans, together with two virgin individuals of the opposite sex from the reference population, as well as one sterilized competitor of the same sex from the reference population. Importantly, all reference individuals were raised in the ancestral environment (29°C on black‐eyed beans), so that putative developmental sensitivity to the novel environments could be attributed solely to the sex and evolution regime of the focal individual. Hence, all assays of competitive LRS had to be performed in the ancestral environment in the adult stage. The presence of a sterilized competitor ensured that focal individuals competed for mating opportunities, as well as postmating fertilization success in the case of males, and egg‐laying sites in the case of females, while all emerging offspring in an assay could be attributed to the focal individual (Eady, 1991; Grieshop et al., 2016; Maklakov & Arnqvist, 2009; Martinossi‐Allibert et al., 2017). Sterilization was achieved by exposing reference individuals to gamma radiation at a dose of 100 Gy, which leaves them sterile for their entire lifetime, while leaving no noticeable effects on competitiveness (Grieshop et al., 2016). Individuals were left to interact during their entire lifetime. After emergence of all offspring of the next generation (following 35 days since the start of the assay), Petri dishes were frozen at −20°C for at least two days before the offspring were counted.

### 2.8 Fertility, phenotypes, and population fitness

These assays were performed on two replicate lines from each evolution regime, maintained at the two temperature conditions. Duplicates were made of each of the six lines and split among the two temperatures. Virgin individuals were collected from each line and used in fertility assays and population fitness assays (20 replicates per assay type, line, and temperature). Here, as we were aiming to score the temperature sensitivity of each evolution regime as a whole (a combined estimate for conspecific males and females), in monogamous single pair settings (fertility) and multiple mating population settings, both assay types could be carried out at respective temperature and did not have to be limited to juvenile development. Body weight, male ejaculate weight, and locomotor activity were measured for the individuals that were used in monogamous assays in order to estimate covariation between fertility and phenotypes. We note that female body weight and male locomotor activity have previously been shown to be genetically correlated to fecundity in the stock population (Berger, Martinossi‐Allibert et al., 2016; Berger, You et al., 2016).

Fertility was measured by counting the lifetime adult offspring production of a female after a single monogamous mating with a randomly assigned male from her own population. This fertility measure thus incorporates the potential fecundity of the female, the fertility of the male, as well as potential effects of the female–male mating interaction. After the mating event, the male and female were separated, preventing further male–female interactions. Female body weight was measured prior to mating and male body weight both before and after mating to estimate the weight of the transferred ejaculate. After mating, females were placed in 6‐cm Petri dishes containing ad libitum (ca 40) black‐eyed beans and left to lay eggs, and males were scored for their locomotor activity.

Male locomotor activity was scored ca. 30 min after the mating event. Twenty males per replicate line per temperature were placed in groups of four in 6‐cm Petri dishes on a heating plate that maintained the original assay temperature (29 or 36°C). After ten minutes of acclimation, each dish was observed every 30 s for 10 min and considered active if one or more of the four individuals were moving (see: Berger, You et al., 2016).

Population fitness was measured at each respective temperature as the average female offspring production in a group of 10 individuals with equal sex‐ratio, placed together in a large Petri dish (6 cm wide, 2 cm deep) with ad libitum (ca. 200) black‐eyed beans and left to interact for their lifetime.

### 2.9 Statistics

#### 2.9.1 Competitive LRS, fertility, and population fitness

Our analyses used maximum‐likelihood estimation from general linear mixed‐effects models, assuming normally distributed data, implemented in the lme4 package (Bates, Maechler, & Bolker, 2011) for R (R Core Team, 2013). Evolution regime, assay environment, and sex and their interactions were specified as fixed effects. Line identity (unique replicate line ID) crossed by environment and sex, as well as date of the assay, were specified as random effects. This thus ensured that the error variance between replicate lines within evolution regimes was used to evaluate statistical significance of all terms including evolution regime, and this general procedure was used in all analyses. When analyzing fertility, evolution regime, assay environment, and male ejaculate weight were specified as fixed effects. Line identity crossed by temperature was specified as a random effect. Population fitness was analyzed in a model including the fixed effects of evolution regime, temperature and their interaction, and line identity crossed by temperature as a random effect. The normality of residuals was checked for all models. For all models, we applied planned contrasts testing for pairwise differences among the three selection regimes following the a priori predictions presented in Table 1.

#### 2.9.2 Female and male weight, ejaculate weight, and male activity

Body weight was analyzed using a linear mixed model assuming a normal distribution. Evolution regime, temperature, and sex and their interactions were specified as fixed effects and line identity crossed by temperature as random effects. Ejaculate weight was analyzed using the same model structure but for the main effect of sex. Finally, activity was analyzed using the same model structure as ejaculate weight but assuming a binomial distribution for the response.

#### 2.9.3 Cost of sociosexual interactions

The change in per female offspring production (B) between monogamous fertility assays and population assays should capture the effect of sociosexual interactions on female viability and reproduction. This change was calculated in relative terms as: 1 − B_population_/B_fertility_.

To estimate B_population_ and B_fertility_, we ran a Bayesian mixed‐effects model implementing Markov Chain Monte Carlo simulations using the MCMCglmm package (Hadfield, 2010) for R. Offspring count was the normally distributed response variable, with temperature, evolution regime, and type of assay (fertility or population fitness) and their interactions as fixed effects. Line identity crossed by temperature and assay type was specified as random effects. Output of the model can be found in Supporting Information Table S2. Default weak priors were used (Variance initiated at 1 and belief set to 0.002 for all random effects) and the number of iterations was set to 1,100,000, of which the first 100,000 were used for burn‐in and later discarded. We stored every 1,000th simulation, resulting in 1,000 uncorrelated posterior estimates upon which we could calculate Bayesian 95% credible intervals for all parameter estimates and *p*‐values for all comparisons.

We extracted posterior distributions for the mean offspring count (B) of each assay type (fertility or population fitness). We then used these posterior distributions to estimate the cost of sociosexual interactions (1 − B_population_/B_monogamy_) for each evolution regime and temperature combination. We focused our effort on testing two hypotheses: (a) that the Male‐limited regime would show a higher cost of sociosexual interactions than the other two regimes (Table 1), and (b) that the increased temperature tolerance of males from the Male‐limited lines (see Results) would make this difference in cost between regimes more pronounced at elevated temperature. To assess whether these hypotheses, we calculated the number of times the difference between posterior estimates of the cost in two groups being compared overlapped zero, with ≤2.5% cases implying statistical significance given a two‐sided hypothesis. The posterior distributions are reported in Supporting Information Table S3 with posterior mode and 95% credible intervals.

## 3 RESULTS

### 3.1 Competitive lifetime reproductive success of males and females

**Figure 1:**
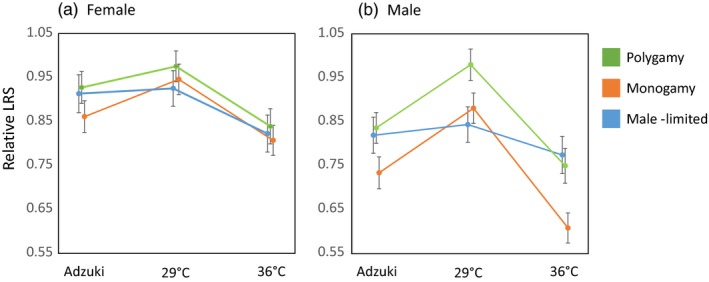
Sex‐specific competitive lifetime reproductive success (LRS) in each of the three assay environments in the three evolution regimes. Female (a) LRS and male (b) LRS were standardized separately by mean LRS of the Polygamy regime at 29°c. Error bars represent one standard error

First, we looked for general differences between the three evolution regimes averaged over the three environments and two sexes by running a model without interaction terms included. This showed that there was a significant difference between the regimes ($\chi_{2}^{2}$= 7.62, *p* = 0.022, Figure 1, Supporting Information Figure S4). This difference was due to the Polygamy regime having higher LRS than the Monogamy regime (Planned comparisons: Polygamy–Monogamy: *z* = 2.75, *p* = 0.0059, Polygamy–Male‐limited: *z* = 1.15, *p* = 0.25, Monogamy–Male‐limited: *z* = −1.41, *p* = 0.16), suggesting that the addition of sexual selection and male–female coevolution was important in maintaining high lifetime reproductive success (Figure 1, see predictions: Table 1).

However, when running the main model with interaction terms added, a significant three‐way interaction including sex, assay environment, and evolution regime suggested that the regimes responded differently to environmental stress and that this difference was sex‐specific ($\chi_{2}^{4}$ = 11.8 *p* = 0.019, Table 2a, Figure 1). Moreover, consistently across all three evolution regimes, males were more affected than females by environmental stress (interaction: $\chi_{2}^{2}$ = 18.0, *p* < 0.001, Table 2a). To examine this further, we ran separate models for the sexes. This showed that the evolution regime by environment interaction was not found in females ($\chi_{2}^{4}$ = 1.35, *p* = 0.85, Table 2b) but present in males ($\chi_{2}^{4}$ = 22.7, *p* < 0.001, Table 2c). This is explained by males from the Male‐limited evolution regime being overall relatively less affected by temperature or host plant than males from the other two regimes (Figure 1b). This suggests that when sexual selection on males acted alone, alleles conferring environmental robustness were enriched relative to the other regimes that applied fecundity selection. Moreover, the increased environmental robustness in the male‐limited regime was limited to males (Figure 1). In females, there was no significant overall effect of evolution regime on competitive LRS (Table 2b), with a marginally nonsignificant difference between the Monogamy and Polygamy regimes (Planned comparisons: Polygamy–Monogamy: *z* = 1.93, *p* = 0.054, Polygamy–Male‐limited: *z* = 0.54, *p* = 0.59, Monogamy–Male‐limited: *z* = −1.21, *p* = 0.23).

**Table 2:** (a) ANOVA table for a general linear mixed‐effect model of competitive lifetime reproductive success, showing the effect of sex, assay environment, and evolution regime and their interactions. (b) and (c) shows the analysis for females and males, respectively. *p*‐values were calculated using type III sums of squares

Fixed effect	*χ* ^2^	*df*	*p*‐value
(a)			
Evolution regime	1.71	2	0.43
Environment	15.7	2	<0.001
Sex	0.45	1	0.5
Environment: Evolution regime	1.85	4	0.76
Sex: Evolution regime	0.67	2	0.72
Sex: Environment	18.0	2	<0.001
Sex:Environment: Evolution regime	11.8	4	0.02
(b)			
Evolution regime	3.84	2	0.15
Environment	14.6	2	<0.001
Environment: Evolution regime	1.35	4	0.85
(c)			
Evolution regime	5.2	2	0.07
Environment	85.2	2	<0.001
Environment: Evolution regime	22.7	4	<0.001

Next, we explored the consequences of evolution under the alternative mating regimes and the observed sex‐specific temperature tolerance for fertility and population fitness.

### 3.2 Fertility of male and female mating pairs

**Figure 2:**
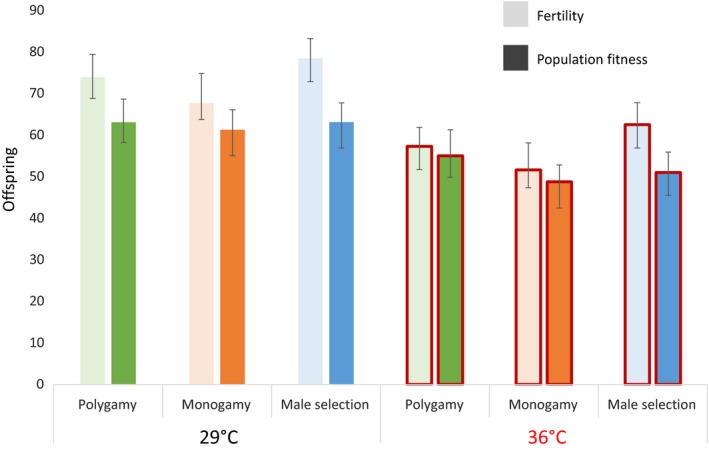
Bayesian posterior modes and 95% credible intervals for fertility (light bars) and population fitness (dark bars) at benign and elevated temperature in the three evolution regimes

Fertility was measured as the lifetime offspring production of females following a single monogamous mating with a conspecific male. Evolution regimes differed in terms of fertility ($\chi_{2}^{2}$ = 8.91, *p* = 0.012, Supporting Information Table S5), an effect that was driven, surprisingly, by the Male‐limited evolution regime having higher fertility than the Monogamy regime (Figure 2, Planned comparisons: Male‐limited–Monogamy: *z* = 2.98 *p* = 0.0028, Polygamy–Monogamy: *z* = 1.44, *p* = 0.15, Polygamy–Male‐limited: *z* = −1.54, *p* = 0.12). Being exposed to elevated temperature decreased fertility across all regimes, but there was no significant interaction between regime and temperature (Supporting Information Table S5a, Figure 2).

To provide insights into the differences in fertility across evolution regimes, we performed an additional analysis on fertility where we added the three covariates to the model: female weight, male weight, and ejaculate weight (see: Supporting Information Table S5b). Female weight was strongly positively related to fertility (Supporting Information Table S5b, $\chi_{2}^{2}$ = 33, *p *= <0.001, Supporting Information Figure S6). However, evolution regimes did not show any significant differences in weight for either sex (Supporting Information Table S7a, Supporting Information Figure S8) that could explain the observed differences in fertility. Ejaculate weight and male size showed no significant effects on fertility (Supporting Information Table S5b).

### 3.3 Population fitness

Population fitness was measured as the offspring production per female in a small population of ten individuals with equal sex‐ratio. Even though the Male‐limited regime had significantly higher fertility than the Monogamy regime, population fitness did not differ significantly between the three regimes overall ($\chi_{2}^{2}$ = 5.24, *p* = 0.073, Supporting Information Table S9 and Figure 2), and the only significant difference was between the Polygamy and the Monogamy regime (Planned comparisons: Polygamy–Monogamy: *z* = 2.30, *p* = 0.022, Male‐limited–Monogamy: *z* = 1.0, *p* = 0.32, Polygamy–Male‐limited: *z* = 1.29, *p* = 0.20). These results are consistent with population fitness being determined by a balance between “good‐genes” sexual selection and sexual conflict, both of which were presumably higher in the Male‐limited regime than in the Monogamy regime, resulting in no obvious difference in population fitness between the two despite clear differences in fertility (Figure 2). Temperature stress significantly reduced population fitness in all evolution regimes ($\chi_{1}^{2}$ = 115, <0.001 Supporting Information Table S9), with no statistically significant interaction between evolution regime and temperature ($\chi_{2}^{2}$ = 3.41, *p* = 0.18, Supporting Information Table S9, Figure 2).

### 3.4 The net cost of sociosexual interactions

**Figure 3:**
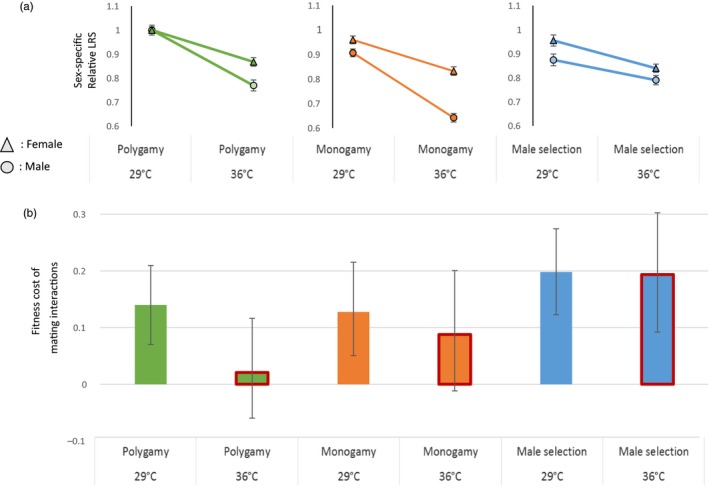
Sex‐specific sensitivity to elevated temperature and associated costs of sociosexual interactions for the three evolution regimes. (a) Relativized competitive LRS is presented separately for each evolution regime to emphasize sex‐specific robustness to elevated temperature in each regime. Error bars represent standard errors. (b) The cost of sociosexual interactions was calculated as *1‐(population fitness/fertility) *and represents the relative drop in offspring production per female between a single monogamous mating and a polygamous group setting from Bayesian posterior modes and 95% credible intervals

The cost of sociosexual interactions was estimated as one minus the ratio of offspring produced per female in the population setting relative to the fertility of monogamous pairs (1 − B_population_/B_fertility)_, thus giving the proportion of offspring lost per female due to sociosexual interactions. This cost, averaged over all three evolution regimes and both temperatures, was greater than 0 in all 1,000 Bayesian simulations (Figure 3, Supporting Information Table S3). There did, however, tend to be differences in this cost across temperatures and evolution regimes (Figure 3). According to our predictions in Table 1, the costs of sociosexual interactions were pronounced in the Male‐limited regime. The Male‐limited regime incurred a greater cost than the Polygamy regime averaged across temperatures (in 995 out of 1,000 simulations, two‐sided Bayesian P_MCMC_ = 0.01, Figure 3, Supporting Information Table S3). This result demonstrates that experimental evolution of male adaptations under sexual selection without female coevolution can confer strong costs at the population level. There was also a tendency for the cost of sociosexual interactions to be greater in the Male‐limited regime compared to the Monogamy regime, as expected if males evolving under sexual selection are more harmful to females than monogamous males, but the difference was marginally nonsignificant (959 out of 1,000 simulations, two‐sided Bayesian P_MCMC _= 0.08, Supporting Information Table S3).

There was an overall tendency for elevated temperature to reduce the cost of sociosexual interactions (942 out of 1,000 simulations). However, the effect of temperature was not consistent across regimes (Figure 3, Supporting Information Table S3). In the Polygamy and Monogamy regimes, the cost appeared to be reduced at elevated temperature, although only markedly so for Polygamy (966 out of 1,000 simulations, two‐sided Bayesian P_MCMC _= 0.07). In contrast, the cost of sociosexual interactions in the Male‐limited regime remained high and constant. The elevated temperature magnified the difference in the cost between the Polygamous and Male‐limited evolution regime (14% vs. 19% at 29°C, *p* = 0.13; −0.01% vs. 19% at 36°C, P_MCMC _= 0.02, Supporting Information Table S3). In Figure 3, we present this result in parallel with the sex‐specific temperature sensitivity of LRS in order to map out the relationship between individual lifetime reproductive success in the two sexes and the effect of sociosexual interactions on population fitness.

In an attempt to unveil the mechanisms and phenotypes mediating the costs of sociosexual interactions, we revisited our data on individual male and female traits measured in the fertility assays. While male and female body weight both increased at elevated temperature ($\chi_{2}^{2}$ = 35.2, *p* < 0.001, Supporting Information Table S7a, Figure S8), we could not reveal any interactions between evolution regime and sex or temperature, suggesting that sex‐specific responses in body weight do not explain putative variation in the cost of sociosexual interactions. Similarly, ejaculate weight was not affected by either temperature or evolution regime (Supporting Information Table S7b). Finally, male locomotor activity, which gives an indication of male harassment in seed beetles (Berger, Martinossi‐Allibert et al., 2016), did not differ between evolution regimes overall, and while male activity decreased at elevated temperature ($\chi_{2}^{2}$ = 5.80, *p* = 0.016, Supporting Information Table S7c, Figure S10), this decrease was similar in all evolution regimes.

## 4 DISCUSSION

Evolution with sexual selection conferred high fertility and lifetime individual reproductive success in both sexes across environments, in line with “good‐genes” effects. However, there was also a substantial cost of sociosexual interactions (indicating IeSC), which counteracted the increase in fertility associated with sexual selection. Indeed, this cost was significantly lower in the Polygamous relative to the Male‐limited regime, highlighting the importance of male–female coevolution in mitigating the impact of IeSC. In the Male‐limited regime, where only sexual selection had been operating, males showed greater environmental robustness than females to both host plant and temperature stress, which was not the case in the Polygamy and Monogamy regime including fecundity selection. Moreover, IeSC seemed to be modulated by this sex‐specificity: The cost of sociosexual interactions tended to be reduced at elevated temperature in the Polygamous and Monogamous evolution regimes, but was maintained at a high level in the Male‐limited evolution regime. Our results thus suggest that (a) sexual selection can lead to evolutionary increases in female fitness components across environments, but that (b) sexual selection also can favor male adaptations that are harmful to females and increase IeSC, and (c) provide proof‐of‐concept for the idea that sex‐specific selection can generate sex‐specificity in environmental robustness, which in turn may modulate sexual conflict and demography of maladapted populations.

The impact of sexual selection on adaptation results from a balance between the benefits of good‐genes effects and costs of sexual conflict. If these processes are affected by environmental change, this balance could be shifted in maladapted populations. However, exactly how these effects will be manifested in novel environments remains unknown because of the lack of empirical data, and here, we therefore tried to explore these dynamics. We used sex‐limited experimental evolution to disentangle the respective contributions of sexual selection, fecundity selection, and male–female coevolution, to individual‐level and population‐level fitness. We then contrasted these effects in well‐adapted populations raised at ancestral conditions, and maladapted populations raised at elevated temperature or on suboptimal hosts. Our study demonstrates how sex‐specific selection can affect the link between individual‐level (mal)adaptation and population viability in polygamous species.

### 4.1 Good genes, environmental robustness, and genotype‐by‐environment interactions

Whether sexual selection generally results in good‐genes effects (Bonduriansky & Chenoweth, 2009; Tomkins et al., 2004), and whether such effects persist in novel environments (Bussiere et al., 2008; Kolluru, 2014; Radwan, 2008), remains a matter of considerable debate. In our experiment, the Male‐limited evolution regime showed the highest fertility of all three evolution regimes, and a female lifetime reproductive success similar to the Monogamy regime (Figure 2), suggesting that sexual selection on males can indeed increase female fitness components in *C. maculatus*. In this species, energetically costly interference and scramble competition are intense (Maklakov & Arnqvist, 2009; Savalli & Fox, 1999), making it likely that males of high quality that carry “good genes” are favored by sexual selection (Whitlock & Agrawal, 2009). In addition to good‐genes effects, there are other, nonmutually exclusive, mechanisms that may have contributed to the high female reproductive output observed in both Polygamy (LRS measure) and Male‐limited lines (fertility measure). For example, manipulation of female physiology by males during mating (e.g., mediated by seminal fluid proteins) could have evolved or been enhanced by male competition under sexual selection. Indeed, such effects are known from other model systems such as fruit flies and nematodes (Chapman, Liddle, Kalb, Wolfner, & Partridge, 1995; Gems & Riddle, 1996).

Males from the male‐limited evolution regime showed high resilience to both host plant and temperature stress, suggesting that sexual selection on males may lead to environmentally robust phenotypes that perform well across environments, rather than locally adapted specialists (see also: Parrett & Knell, 2018). Interestingly, however, males from the polygamous regime (that also applied sexual selection) did not show the same environmental robustness. This suggests that a balance between fecundity selection and sexual selection is important in shaping sex‐specificity in environmental robustness and may be central in maintaining alternative alleles encoding this trait in *C. maculatus* populations. Such alleles are likely to contribute to adaptation in new environments, supporting the idea that opposing forces of natural and sexual selection can maintain genetic variation that may fuel adaptive responses to environmental change (Radwan, Engqvist, & Reinhold, 2016).

### 4.2 sociosexual interactions and population demography upon environmental change

Despite leading to genetic increases in female fitness components, we also saw that sexual selection can favor individual male strategies that bear costs at the population level. We observed a cost of sociosexual interactions in all evolution regimes at the ancestral temperature (Figure 3). We suggest that this effect is mainly mediated by IeSC, given that there are well‐known costs to females of mating multiply and documented sexually antagonistic coevolution involving male and female genitalia in this species (Crudgington & Siva‐Jothy, 2000; Dougherty et al., 2017; Edvardsson & Tregenza, 2005; Gay et al., 2011; Rönn, Katvala, & Arnqvist, 2007). Our study also suggests that IeSC may evolve to become magnified under sexual selection when female counteradaptation is constrained from mitigating the harm incurred by male mating strategies (Rice, 1996), supported by the higher cost of sociosexual interactions in Male‐limited relative to Monogamous and Polygamous lines. This result is consistent with theoretical models suggesting that sexual selection can lead to the evolution of male traits that are harmful to females and thereby can contribute to population extinction (Kokko & Brooks, 2003; Rankin et al., 2011). This view is also in agreement with recent evidence from the fossil record showing that lineages of ostracods with higher sexual dimorphism, as a correlate of the strength of sexual selection and conflict, have higher rates of extinction (Martins, Puckett, Lockwood, Swaddle, & Hunt, 2018).

The potential impact of IeSC on population viability has received considerable attention (Arnqvist & Rowe, 2005; Clutton‐Brock & Parker, 1995; Parker, 1979, 2006; Thornhill & Alcock, 1983), and recent empirical studies have explored its effects across variable ecological conditions (Arbuthnott et al., 2014; Berger, Olofsson, Gotthard, Wiklund, & Friberg, 2012; den Hollander & Gwynne, 2009; Garcia‐Roa, Chirinos, & Carazo, 2018; Gay, Eady, Vasudev, Hosken, & Tregenza, 2009; Gomez‐Llano et al., 2018; Iglesias‐Carrasco, Jennions, Zajitschek, & Head, 2018, MacPherson, Yun, Barrera, Agrawal, & Rundle, 2018; Rowe & Arnqvist, 2002; Sakurai & Kasuya, 2008; Takahashi, Kagawa, Svensson, & Kawata, 2014; Takami, Fukuhara, Yokoyama, & Kawata, 2018; Yun et al., 2018). Some of the more recent studies highlight a particular role for environmental complexity in mediating IeSC and its consequences (MacPherson et al., 2018; Yun et al., 2018). Above and beyond that, however, making predictions about the extent and change in IeSC and its consequential impact on population demography upon environmental change is complicated by the inherent unpredictability of environmental change itself. For example, it has been argued that IeSC may be reduced under low population density (Arnqvist, 1992; Gerber & Kokko, 2016), as can be expected in declining populations suffering from maladaptation to local environmental conditions. In this case, IeSC would be relaxed and the population relieved of the sexual conflict load. However, depending on the context driving population decline, it remains uncertain whether general declines in numbers of breeding pairs will result in lower densities at mating sites, especially if the drivers of population decline are factors like degradation and fragmentation of suitable breeding habitats, which may instead result in higher densities of reproducing adults.

Here, therefore, we raised one possible heuristic scenario that could generate predictable changes in IeSC and its demographic cost upon environmental change. If environmental stress affects one sex more than the other (e.g., because of different resource use: Maklakov et al., 2008; Zajitschek & Connallon, 2017), IeSC could either be intensified or reduced depending on which sex is the most sensitive to the change in ecological conditions (Clutton‐Brock & Parker, 1995; Rankin et al., 2011). Interestingly, the greater female bias in environmental robustness found in the Polygamy and Monogamy regime, relative to the Male‐limited regime where fecundity selection was removed, suggests that the balance between natural and sexual selection may shape sex‐specific environmental robustness. As follows from our hypothesis, these sex differences in environmental robustness seemed to be accompanied by parallel changes in the cost of sociosexual interactions at elevated temperature, with lowered costs observed in the Polygamy regime, but maintained costs in the Male‐limited regime (Figure 3). This thus raises the possibility that sex‐specific selection can lead to sex‐specificity in environmental robustness, which in turn can modulate the cost of sexual conflict. While this principle may be general, it remains to be explored how great its effect is in natural populations and how predictable sex differences in environmental robustness are in naturally variable environments. Both these aspects, along with the fact that multiple mating also may provide females with benefits that are likely dependent on the condition of the male (Arnqvist & Nilsson, 2000), will need to be understood in order to forecast the demographic impact in changing environments.

### 4.3 Traits underlying changes in the cost of sociosexual interactions

In an attempt to better understand how the relationship between sex‐specific environmental robustness and IeSC can be generalized to other animals, we investigated putative traits that may have mediated the observed costs of sociosexual interactions. Body mass often reflects phenotypic condition, and sex differences in body mass can modulate the intensity of IeSC (Clutton‐Brock & Parker, 1995). Indeed, sexual size dimorphism is likely to be related to the amount of male‐inflicted harm on females as it likely affects both the ability of males to coerce females and the females’ ability to cope with harmful male mating behaviors (Arnqvist & Rowe, 2005). Hence, because the juvenile environment is known to affect sexual size dimorphism in many species (Stillwell, Blanckenhorn, Teder, Davidowitz, & Fox, 2010), IeSC may also change across environments. In the present study however, body mass did not explain the cost of sociosexual interactions. The argument could be extended to other putatively condition‐dependent traits that are involved in IeSC. Locomotor activity, which is related to male courtship activity in many species (Arnqvist & Rowe, 2005, for *C. maculatus*: Gay et al., 2009), was lower at elevated temperature. However, male activity did not differ significantly between evolution regimes, suggesting that it was not the trait responsible for the maintained cost of sociosexual interactions at elevated temperature in the Male‐limited regime. Interestingly, Garcia‐Roa et al. (2018) recently demonstrated that IeSC is reduced at elevated temperature in *D. melanogaster*, and in their study, the relative rates of male mating attempts and female rejections of males between high and low sexual conflict settings did show changes across temperatures concomitant with the changes in IeSC. The general findings in these two studies on two different insect models for sexual selection, suggesting that elevated temperature may reduce IeSC in naturally polygamous populations, suggest that the observed pattern could be widespread, and it would be interesting to explore its generality across other ectothermic taxa.

Finally, contrary to what has been observed in insect taxa like *D. melanogaster* (Lung et al., 2002; Mueller et al., 2007; Wigby & Chapman, 2005), we found no support for ejaculate toxicity mediating IeSC to the extent that ejaculate weight did not vary across evolution regimes. We do note that this does not rule out the possibility that the composition of the seed beetle ejaculate (Goenaga, Yamane, Rönn, & Arnqvist, 2015; Vasudeva, Deeming, & Eady, 2014) may have played a role in generating variation in both fertility and IeSC across temperatures and evolution regimes.

## 5 CONCLUSIONS

In summary, our study points to multiple facets by which sexual selection can contribute to either evolutionary rescue or extinction of maladapted populations. Our results highlight that these effects can be interdependent because sexual selection on males can (a) elevate fertility of females via good‐genes effects, but also (b) intensify sexual conflict, and (c) involve loci with environment‐specific effects, affecting direct as well as indirect genetic responses in novel environments, and finally (d) affect sex‐specific environmental robustness, which in turn may modulate the intensity of sexual conflict in maladapted populations.

## CONFLICT OF INTEREST

None declared.

## DATA ARCHIVING

Data available from the Dryad Digital Repository: https://doi.org/10.5061/dryad.77c4555.
