# Supplementary material for: Sexual selection, environmental robustness, and evolutionary demography of maladapted populations: A test using experimental evolution in seed beetles
Source: Evol Appl. 2019 Feb 19;12(7):1371–84. doi: 10.1111/eva.12758 (PMC6691221; doi:10.1111/eva.12758)

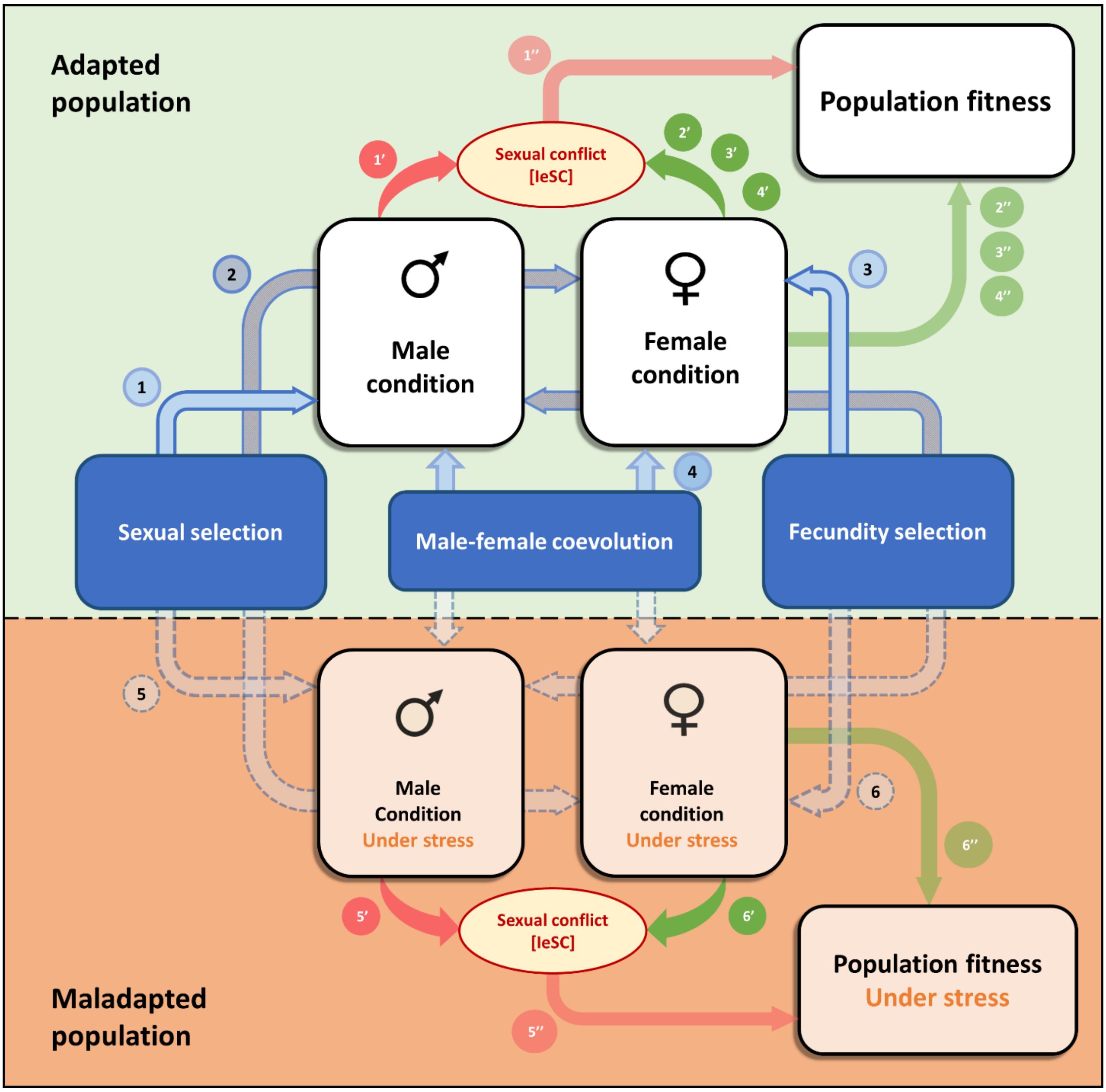

Supplementary figure S1: Predicted effects of sexual selection, fecundity selection and male-female coevolution on fitness of well-adapted and maladapted populations.

Sexual selection, fecundity selection and male-female coevolution may all affect male and female condition independently, modulating both the reproductive potential of each sex as well as the intensity of interlocus sexual conflict (IeSC). These processes, and particularly the

resulting reproductive output of females, are expected to impact population fitness, but their dynamics could be modified upon rapid environmental change. Here we highlight some main scenarios for how individual-level selection on males and females is expected to affect population-level fitness.

-In a well-adapted population (green panel):

Sexual selection may increase male condition (1), resulting in intensified IeSC and reduced female fecundity (1') and population fitness (1"). It may also affect female condition (2), either positively by generally reducing mutation load, resulting in increased population fitness (2"), or negatively under pronounced intralocus sexual conflict (IaSC). Fecundity selection is expected to elevate female condition (3), which should increase population fitness (3"). Moreover, male-female coevolution (4) may allow females to evolve resistance to harmful male reproductive tactics, reducing the negative impact of IeSC on female fecundity (4') and population fitness (4").

-In a maladapted population (orange pannel):

A history of sexual selection and fecundity selection could either have favored individuals that are generally resistant to stress, resulting in improved condition also in the novel environment, or have favored locally adapted genotypes, resulting in those genotypes having low fitness in the novel environment (5, 6). In particular, how previous sexual selection in males (5) and fecundity selection in females (6) affects the expression of female condition in the novel environment is predicted to have direct consequences for population viability in maladapted populations (6"). While stress-resistant genotypes are predicted to result from purifying "good genes" sexual selection against deleterious mutations, genotype-by-environment interactions have often been observed for male reproductive traits, suggesting that sexual selection in one

environment may not necessarily result in increased condition in another. Moreover, if sexual selection and fecundity selection acts in fundamentally different ways to affect the expression of male (5’) and female (6’) condition in novel or stressful environments, this could lead to either an increased (5’ > 6’) or decreased (5’ < 6’) impact of IeSC on the viability of maladapted populations (5’’, 6’’) as a result of changes in the relative condition of male and female genotypes.

Supplementary table S2. Output of the MCMCglmm model used to estimate the cost of socio-sexual interactions. The response variable was number of offspring; evolution regime, temperature and type of assay (population fitness or fertility assay) as well as their interactions were specified as fixed effects. Evolution replicate, temperature by replicate and assay type by replicate were specified as random effects.

**Fixed Effect**	**Posterior mean**	**95% CI**	**P_{MCMC}**
Intercept	62.45	56.03/68.27	<0.001
Assay type: Fertility	15.73	9.38/22.02	<0.001
Selection: Monogamy	-1.89	-12.17/7.06	0.67
Selection: Polygamy	0.98	-7.52/10.97	0.81
Temperature: 36C	-11.7	-19.12/-3.99	0.01
Fertility by Monogamy	-7.19	-16.64/1.87	0.13
Fertility by Polygamy	-5.15	-15.31/3.31	0.25
Fertility by 36C	-3.67	-11.85/4.76	0.38
Monogamy by 36C	-0.59	-12.13/9.98	0.92
Polygamy by 36C	3.7	-7.10/15.51	0.46
Fertility by Mongamy by 36C	-0.71	-11.73/12.52	0.9
Fertility by polygamy by 36C	-5.65	-16.59/6.58	0.34
**Random effects**			
Evolution Replicate	5.92	0.0031/21.0	
Temperature by Replicate	7.76	0.0034/29.1	
Assay type by Replicate	1.75	0.0035/8.0	
Residuals	182.2	160.5/207.5	

Posterior distributions of the cost of mating interactions

29C			
Regime	Posterior mode	Low 95% CI	High 95% CI
Male	0.19	0.13	0.28
Monogamy	0.13	0.037	0.2
Polygamy	0.14	0.063	0.21

36C			
Regime	Posterior mode	Low 95% CI	High 95% CI
Male	0.19	0.094	0.26
Monogamy	0.089	-0.021	0.19
Polygamy	-0.008	-0.082	0.12

Temperatures pooled			
Regime	Posterior mode	Low 95% CI	High 95% CI
Male	0.2	0.14	0.25
Monogamy	0.11	0.031	0.17
Polygamy	0.095	0.017	0.15

Selection regimes pooled			
Temperature	Posterior mode	Low 95% CI	High 95% CI
29C	0.15	0.11	0.2
36C	0.11	0.041	0.15

Overall cost	Posterior mode	Low 95% CI	High 95% CI
	0.12	0.091	0.16

Supplementary table S3. Posterior distributions extracted from a MCMCglmm model for the cost of socio-sexual interactions calculated as 1 - $B_{population}$ / $B_{monogamy}$ were B represents mean offspring production for population assays and fertility, assays respectively. Costs of socio-sexual interactions are given for each evolution regime and temperature combination.

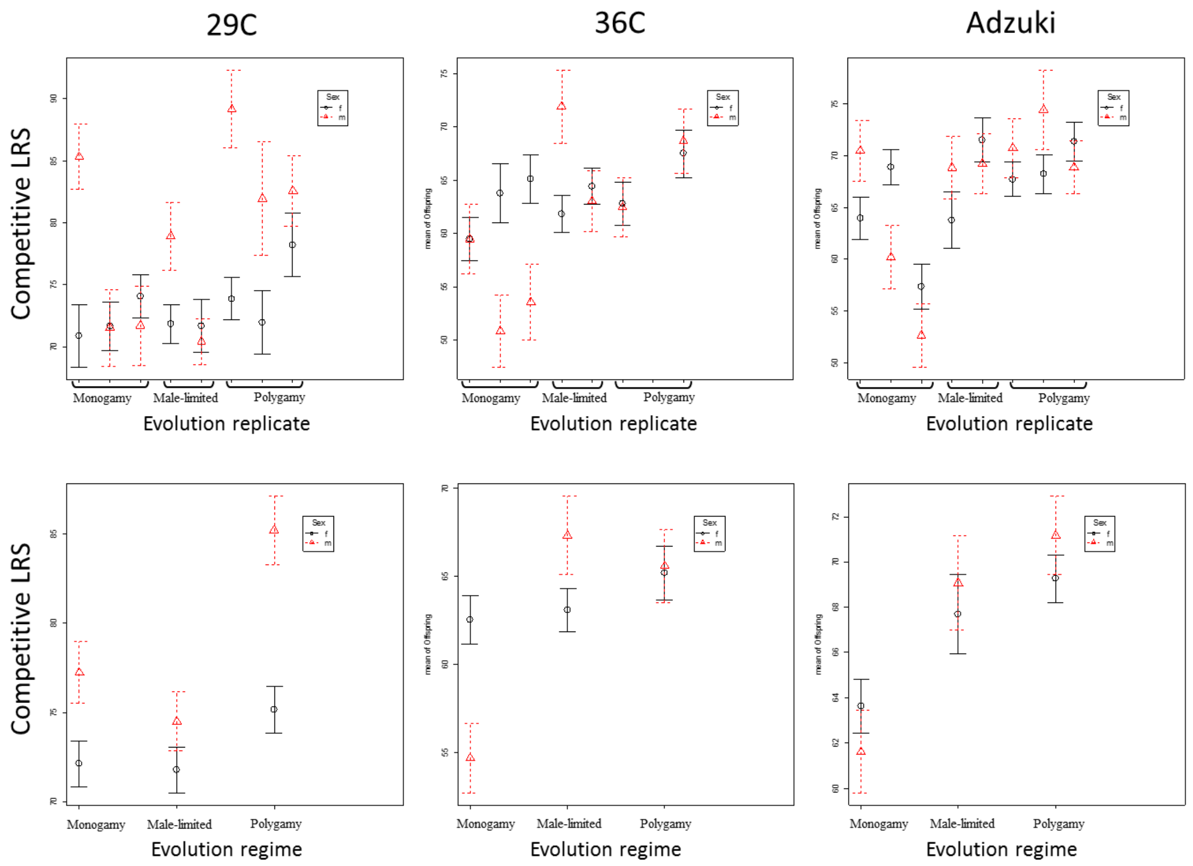

Supplementary figure S4. Mean competitive LRS for each evolution regime (bottom row) and each replicate line of the evolution regimes (upper row), for females (black circles) and males (red triangles).

Supplementary table S5. (a) Anova table for a general linear mixed-effect model of fertility, showing the effect of assay temperature, evolution regime and ejaculate weight. P-values were calculated using type II sums of squares.

(b) Anova table for a general linear mixed-effect model of fertility, showing the effect of assay temperature, evolution regime and ejaculate weight together with female weight and male weight. P-values were calculated using type II sums of squares.

a.

**Fixed Effect**	χ^2	**df**	**P-value**
Evolution regime	8.91	2	0.03
Temperature	39.5	1	<0.001
Ejaculate weight	0.07	1	0.79
Temperature : Evolution regime	0.08	2	0.96

b.

**Fixed Effect**	χ^2	**df**	**P-value**
Evolution regime	12	2	0.002
Temperature	84	1	<0.001
Ejaculate weight	0.19	1	0.67
Female weight	33	1	>0.001
Male weight	0.64	1	0.42
Temperature : Evolution regime	0.59	2	0.75

Supplementary figure S6. The relationship between female weight and fertility across temperatures and experimental evolution treatments.

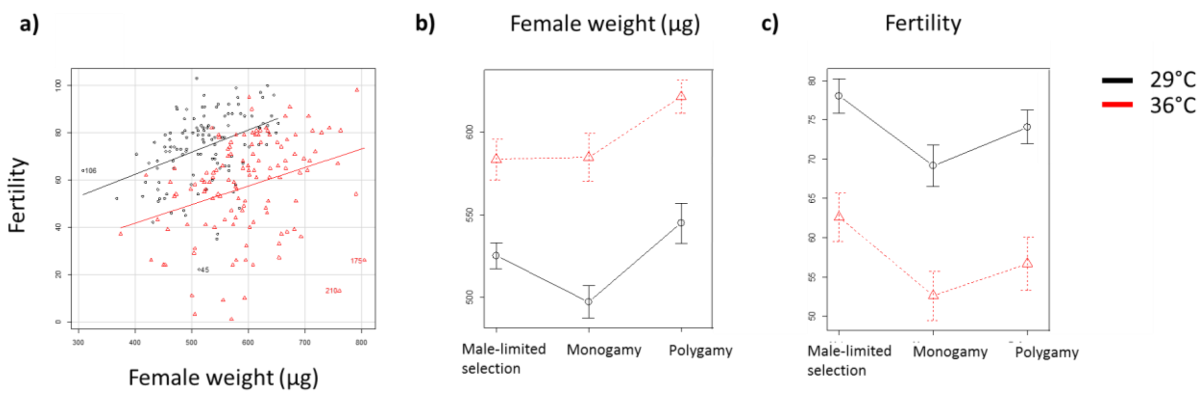

Supplementary table S7.a Anova table for a general linear mixed-effect model on body weight showing the effect of assay temperature, evolution regime, sex and their interactions.

**Fixed Effect**	χ^2	**df**	**P-value**
Evolution regime	4.50	2	0.11
Temperature	35.2	1	<0.001
Sex	1117	1	<0.001
Temperature: Evolution regime	0.784	2	0.68
Sex : Evolution regime	4.26	2	0.12
Sex : Temperature	2.15	1	0.14
Sex : Temperature: Evolution regime	0.0073	2	0.99

Supplementary table S7.b Anova table for a general linear mixed-effect model on male ejaculate weight, showing the effect of assay temperature, evolution regime, and their interactions.

**Fixed Effect**	χ^2	**df**	**P-value**
Evolution regime	1.13	2	0.57
Temperature	0.083	1	0.77
Temperature: Evolution regime	2.72	2	0.26

Supplementary table S7.c Anova table for a generalized linear mixed-effect model on male activity, assuming Binomial errors, showing the effect of assay temperature, evolution regime and their interactions.

**Fixed Effect**	**χ^2**	**df**	**P-value**
Evolution regime	2.01	2	0.37
Temperature	5.80	1	0.016
Temperature: Evolution regime	0.151	2	0.93

Supplementary figure S8. The effect of temperature and evolution regime on female and male body weight.

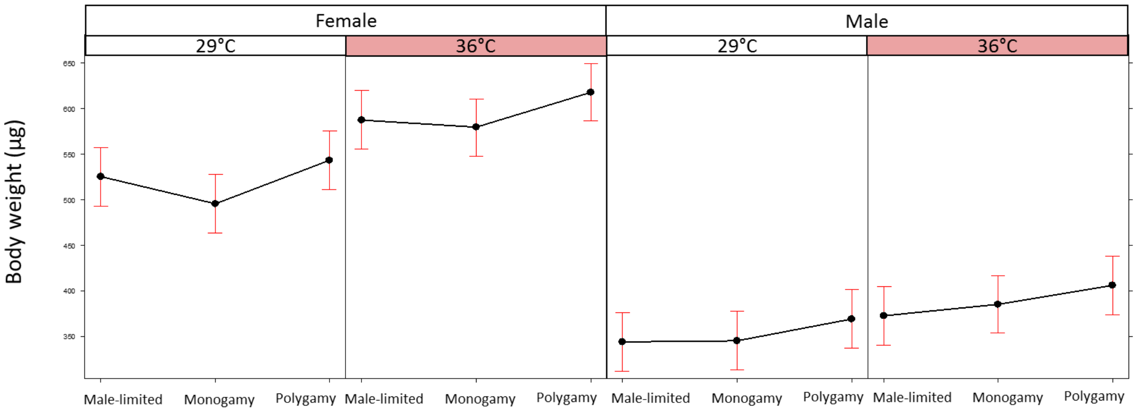

Supplementary table S9. Anova table for a general linear mixed-effect model on population fitness, showing the effect of assay temperature, evolution regime and their interactions.

**Fixed Effect**	χ^2	**df**	**P-value**
Evolution regime	5.24	2	0.073
Temperature	115	1	<0.001
Temperature: Evolution regime	3.41	2	0.18

Supplementary figure S10. The effect of temperature and evolution regime on male activity.

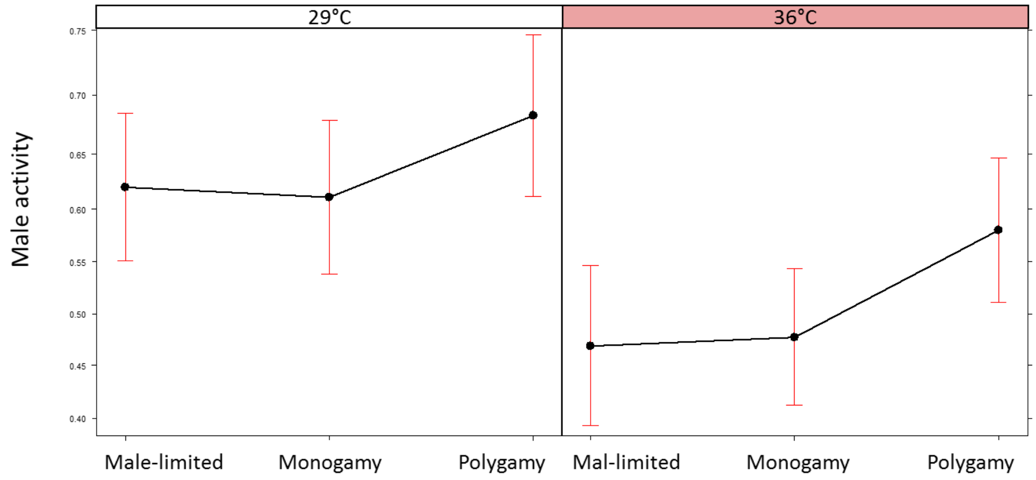

Supplement: Supplementary file 1 [file EVA-12-1371-s001.pdf]
